# Effects of motor imagery brain-computer interface task on quantitative EEG features in patients with prolonged disorders of consciousness

**DOI:** 10.3389/fnins.2026.1815881

**Published:** 2026-05-18

**Authors:** Zhenzhen Gao, Zhiwen Zhu, Shiyan Wang, Yucheng Wu, Zhufeng Song, Yanglu Guo, Haochong Wang, Ya-jun Mao

**Affiliations:** 1 Department of Rehabilitation Medicine, The First Affiliated Hospital of Zhejiang Chinese Medical University (Zhejiang Provincial Hospital of Chinese Medicine), Hangzhou, China; 2 Institute of Health and Rehabilitation Sciences, School of Life Science and Technology, Xi‘an Jiaotong University, Xi'an, China

**Keywords:** brain-computer interface, electroencephalography, motor imagery, prolonged disorders of consciousness, quantitative EEG

## Abstract

**Objective:**

To analyze quantitative electroencephalographic (EEG) characteristics during Motor Imagery Brain-Computer Interface (MI-BCI) task in patients with prolonged disorders of consciousness (pDoC).

**Methods:**

Forty-three patients with pDoC due to various brain injuries were enrolled. Based on modified Coma Recovery Scale-Revised (CRS-R) assessments, the patients were divided into 19 in the unresponsive wakefulness syndrome (UWS) group and 24 in the minimally conscious state (MCS) group. All patients underwent 5 min of resting-state (RS) EEG followed by 5 min of MI-BCI task. Relative power, DTABR, and average brain engagement (BE) during MI-BCI were analyzed across resting and MI-BCI states using Fast Fourier Transform (FFT) spectra.

**Results:**

Mixed-design ANOVA showed significant main effects of condition and group across all EEG frequency bands, indicating clear differences between the RS and MI-BCI conditions and between UWS and MCS patients. Significant group × condition interactions were found in the delta, beta, and gamma bands, as well as in DTABR. Simple effects analysis showed that delta power was higher in RS than in MI-BCI in both groups, with UWS consistently exhibiting higher delta power than MCS under both conditions. In contrast, beta and gamma power were higher in MI-BCI than in RS in both groups. For beta power, UWS was higher than MCS under RS, whereas MCS was higher than UWS under MI-BCI, showing a reversal of the interaction pattern. For gamma power, MCS showed higher values than UWS under both conditions, with a larger between-group difference during MI-BCI. DTABR was significantly higher in RS than in MI-BCI in both groups; however, MCS exhibited higher DTABR than UWS under RS, whereas the opposite pattern was observed under MI-BCI. In addition, during MI-BCI tasks, the MCS group showed greater average BE than the UWS group.

**Conclusion:**

MI-BCI shows potential as a diagnostic or assessment tool for evaluating the level of consciousness in patients with pDoC.

## 1 Introduction

Prolonged disorders of consciousness (pDoC) refer to pathological states characterized by loss of consciousness lasting over 4 weeks due to various brain injuries. These conditions are classified into unresponsive wakefulness syndrome (UWS) and minimally conscious state (MCS) (Giacino et al., 2018). Research (Helbok et al., 2022) indicates that the number of pDoC patients in the United States has exceeded 400,000, while China is seeing an annual increase of 100,000 cases, imposing a heavy burden on patient families and society. To date, no prior study has systematically quantified EEG features recorded during Motor Imagery Brain-Computer Interface (MI-BCI) task in patients with pDoC, leaving a critical knowledge gap in objectively assessing residual cognitive function in this population. Currently, the Coma Recovery Scale-Revised (CRS-R) is considered the gold standard for clinically assessing consciousness levels. However, single behavioral assessments are susceptible to fluctuations in behavior and language barriers. Studies using CRS-R evaluations indicate a misdiagnosis rate of up to 38.2% for MCS (Wang et al., 2020). Accurately assessing the consciousness level of pDoC patients and helping families understand the likelihood of recovery of consciousness often pose significant challenges.

Quantitative electroencephalography analysis holds significant clinical value in assessing consciousness levels and predicting outcomes in patients with impaired consciousness. Research indicates that analyzing patients' neurophysiological responses to auditory or specific paradigm stimuli—such as mismatch negativity (MMN) and P300 event-related potentials—can effectively distinguish between coma, MCS, and UWS and predict the probability of consciousness recovery within 6 months (Shao et al., 2025). Since a significant proportion of patients with impaired consciousness retain latent cognitive abilities (Kondziella and Stevens, 2022), they may possess some capacity for motor imagery. Furthermore, the neural control commands generated during motor attempts or imagery do not depend on the integrity of motor function (Angerhöfer et al., 2021). Non-invasive BCI technology based on EEG signals can decode the electrical activity characteristics of the central nervous system in real time, converting patients' neural control commands into artificial command outputs. This enables interaction between the central nervous system and external devices or environments (Wolpaw et al., 2020). However, current development of EEG-based BCI systems primarily relies on quantitative EEG features derived from healthy subjects or specific patient populations. The closed-loop feedback mechanism inherent to BCI may alter quantitative EEG features by inducing neuroplasticity (Rodríguez-García et al., 2025). This suggests that BCI technology can serve not only as a tool for detecting residual cognitive function in patients with pDoC but also potentially exert therapeutic effects through neuroplasticity mechanisms. Therefore, investigating quantitative EEG characteristics during BCI application in pDoC patients not only helps refine consciousness assessment systems but also provides objective biomarkers for neuromodulation therapies.

This study integrated motor imagery techniques with BCI technology to collect EEG signals from patients with varying levels of consciousness during MI-BCI task. Quantitative analysis of these signals, alongside RS EEG, was conducted to investigate the potential role of MI-BCI in assessing consciousness levels and informing treatment strategies for pDoC patients.

## 2 Materials and methods

### 2.1 Participants

This study was approved by the Ethics Committee of the First Affiliated Hospital of Zhejiang University of Chinese Medicine (2024-KLS-560-02). Patient inclusion criteria were: (1) Age 18–70 years; (2) Confirmed diagnosis of pDoC; (3) Etiology including traumatic brain injury, cerebral infarction, cerebral hemorrhage, hypoxic encephalopathy, etc.; (4) Right-handed individuals. Exclusion criteria included: (1) History of other neurological or psychiatric disorders prior to impaired consciousness; (2) Patients who underwent cranial decompressive craniectomy, cranial repair surgery, or neurostimulator implantation; (3) Use of medications affecting cortical excitability; (4) Presence of uncontrolled epilepsy or involuntary movements; (5) Visual or hearing impairment preventing comprehension of instructions; (6) Family refusal to sign informed consent.

This study recruited 43 subjects meeting the above criteria at the First Affiliated Hospital of Zhejiang University of Chinese Medicine from January 2025 to December 2025. All patients underwent three consecutive CRS-R assessments over 3 days (days 3–5 post-admission) by trained physicians. When assessment results differed, the highest score was recorded. Based on these results, 19 patients were assigned to the UWS group and 24 to the MCS group. Demographic comparisons between groups are presented in Table 1.

**Table 1:** Participant characteristics.

Group	Age (years)	Sex (cases)		Cause (cases)				Duration (days)	CRS-R (score)
		M	F	TBI	ICH	CI	HIE		
UWS	58.34 ± 7.28	10	9	8	5	4	2	67.59 ± 16.73	6 (5, 7)
MCS	60.59 ± 8.92	12	12	9	7	5	3	71.66 ± 18.52	14 (11, 16)
*t/χ^2^*/*U*	−0.990	0.030		2.184				−0.676	128.0
*P*	0.327	0.863		0.536				0.502	< 0.001

M, male; F, female; TBI, traumatic brain injury; ICH, intracerebral hemorrhage; CI, cerebral infarction; HIE, hypoxic-ischemic encephalopathy.

### 2.2 EEG acquisition and MI-BCI task

#### 2.2.1 Resting-state EEG recording

**Figure 1:**
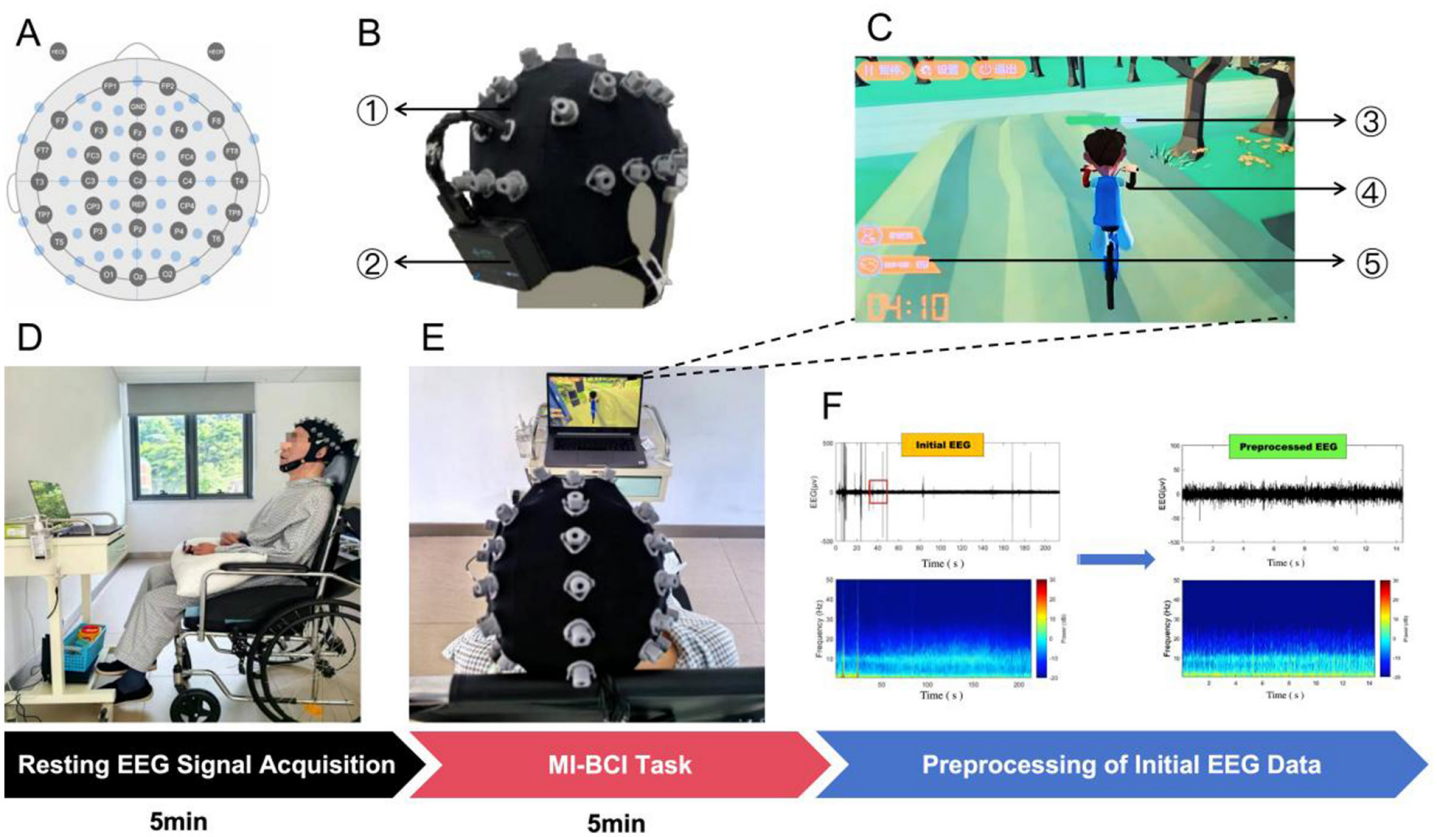
**(A)** 32-channel EEG cap electrode placement layout. **(B)** 32-channel EEG recording cap; Wireless multi-channel EEG signal amplifier. **(C)** MI-BCI human-computer interaction virtual reality scene interface. BE bar; A virtual reality character on a bike ride; BE score. **(D)** The resting EEG recording process. **(E)** MI-BCI virtual reality cycling task. **(F)** Preprocessing of initial EEG data.

EEG signals were acquired in a quiet, softly lit, temperature-controlled indoor environment. A 32-channel wireless multi-channel EEG acquisition and analysis system (Zhen-Tec NT1, Xi'an Zhen-Tec Intelligent Technology Co., Ltd., Xi'an, Shaanxi, China) is used for resting EEG collection. Prior to acquisition, the electrolyte solution is prepared to moisten Lingke cotton pads. The EEG signal acquisition cap (Figure 1B) is assembled and properly fitted on the patient. Electrodes were placed strictly according to the international 10–10 system (Figure 1A). Sampling frequency: 500 Hz. Electrode impedance within 20KΩ. Verify EEG acquisition quality. Collect 5 min of RS EEG data under quiet, interference-free conditions. The acquisition process is illustrated in Figure 1D.

#### 2.2.2 MI-BCI task procedure

After the RS EEG was recorded, the MI-BCI task was initiated. With their eyes open, the patient was presented with a screen displaying a virtual reality scene and a character. They were then given a verbal instruction: “Now imagine that the person on the screen is you. You can start riding.” The 32-channel EEG recording continued throughout this process. Event-related desynchronization of the β rhythm at the Cz electrode triggered the character's cycling motion in the virtual reality environment. The attention intensity feature was calculated using the energy-weighted ratio of the β and α bands at the Fp1 electrode, normalized to an integer value between zero and 100, and defined as Brain Engagement (BE).

The virtual character's cycling speed was controlled in real time via BE: the higher the BE, the faster the cycling speed. All patients underwent training prior to performing the MI-BCI task. If a patient was unable to complete the motor imagery task or exhibited low BE, the avatar remained stationary. During the MI-BCI task, a voice prompt was provided every 10 s: “You're doing great—keep it up!” The task lasts for 5 min, during which behavioral responses, including limb movements and eye-open/eye-closed status, are recorded. The formula for calculating BE is as follows (Yuan et al., 2021):


$$\begin{array}{lll} R & = & \frac{E_{\beta }}{E_{\alpha }}\\ BE & = & MIN+\frac{\left(MAX+MIN\right)\left(R-min\right)}{\max-\min} \end{array}$$

In this formula, *R* represents the beta-to-alpha energy ratio. E_β_ is the total power in the beta band, and E_α_ is the total power in the alpha band. MAX = 100 and MIN = 0 represent the upper and lower limits of the normalization range, respectively. MAX and MIN are empirical constants derived from data calculated during the initial training phase. The task process is illustrated in Figures 1C, E.

### 2.3 EEG data analysis

#### 2.3.1 Data preprocessing

EEG data preprocessing was conducted in MATLAB 2021a (The MathWorks, Inc., Natick, Massachusetts, USA) using the EEGLAB toolbox, as illustrated in Figure 1F. The preprocessing pipeline comprised the following steps:

(1) Electrode localization: electrode coordinate files corresponding to the EEG cap montage were first imported to ensure accurate spatial information for subsequent analyses.
(2) Line noise removal: a notch filter implemented in ERPLAB was applied to attenuate 50 Hz power-line interference.
(3) Band-pass filtering: continuous EEG data were band-pass filtered between one and 80 Hz using a sixth-order Butterworth filter to remove slow drifts and high-frequency noise while preserving the frequency range of interest.
(4) Removal of non-EEG channels: non-cerebral channels, including electrooculography electrodes such as HEOL and HEOR, were excluded from the scalp EEG dataset prior to further analysis.
(5) Independent component analysis and artifact rejection: independent component analysis (ICA) was performed to separate the continuous EEG into temporally independent sources. Artifactual components were identified based on their spatial topography, time-course characteristics, and power spectral features, and were then removed prior to signal reconstruction.

Particular attention was paid to the identification of muscle artifacts, a major concern in high-frequency EEG analyses. Components were classified as muscle-related when they exhibited one or more of the following characteristics: (1) a focal or peripheral scalp distribution, typically concentrated over temporal, frontal, or neck-adjacent electrodes; (2) irregular, short-duration, high-frequency activity in the component time series; (3) a broadband power spectrum with disproportionately elevated power in higher frequency ranges, especially in the beta and gamma bands, without a physiologically plausible neuronal peak; (4) spectral profiles showing a steep increase in high-frequency power or a broad, non-oscillatory high-frequency pattern consistent with myogenic activity. Components identified as muscle artifacts were systematically removed. To minimize the risk of discarding neural signals of interest, component rejection was performed conservatively and only when the artifact profile was unequivocal across multiple criteria.

(6) Manual inspection and channel/segment correction: after ICA-based correction, the data were manually reviewed to identify residual artifacts. Segments showing substantial baseline drift, abrupt high-amplitude fluctuations, or other non-physiological contamination were rejected. Noisy or malfunctioning electrodes were identified through visual inspection and signal quality assessment, and bad channels were subsequently reconstructed using interpolation from neighboring electrodes.

In addition, because EMG contamination can persist after ICA, special care was taken to verify the quality of the cleaned data in the high-frequency range. Specifically, the power spectral density in the gamma band was visually inspected after artifact rejection to confirm the absence of spectral patterns typically associated with residual EMG contamination, such as broadband high-frequency elevation lacking a clear neurophysiological structure.

(7) Re-referencing: finally, the EEG data were re-referenced to the common average reference, whereby the potential at each electrode was recalculated relative to the mean potential across all retained scalp electrodes.

#### 2.3.2 Relative power analysis

The relative power for each frequency band is calculated by dividing the global average of the target frequency band by the total power across the entire analyzed frequency range. Power spectral density analysis of the EEG data was performed using the Welch method with a 1-s time window and 50% overlap to calculate total power. Finally, the ratio of each band's power to the total power was used as its relative power. Where: delta (δ): 1–4 Hz, theta (θ): 4–8 Hz, alpha (α): 8–13 Hz, beta (β): 13–30 Hz, Gamma (γ): 30–80 Hz. Calculation formula:


$$\begin{array}{l} RP\left(f_{1,\;}f_{2}\right)\;=\;\frac{Power\;\left(f_{1},\;f_{2}\right)}{Power\;\left(1,\;80\right)} \end{array}$$

Power (*f*_1_, *f*_2_) denotes the power between frequencies *f*_1_ and *f*_2_, and Power (1, 80) represents the total power in the 1–80 Hz range.

#### 2.3.3 Delta-theta to alpha-beta ratio (DTABR)

Calculated as (delta + theta)/(alpha + beta), this metric reflects the patient's brain activity state and serves as a biomarker of post-injury recovery potential (Olivia et al., 2024).

#### 2.3.4 Average brain engagement

The average BE scores recorded during the MI-BCI task.

### 2.4 Statistical analysis

Statistical analysis was performed using GraphPad Prism 9.0 software (GraphPad Software, LLC, La Jolla, California, USA). Continuous variables were assessed using the Shapiro–Wilk and Levene-tests and met the criteria for normality and homogeneity of variance. The count data were analyzed using chi-square tests. Quantitative data are expressed as (mean ± SD). A 2 × 2 mixed-design analysis of variance (ANOVA) was conducted separately for each frequency band and DTABR, with Group (UWS vs. MCS) as the between-subject factor and Condition (RS vs. MI-BCI) as the within-subject factor. For each band, the main effects of Group and Condition, as well as the Group × Condition interaction, were evaluated. Effect sizes were expressed as partial eta squared$\left(\eta_{p}^{2}\right)$. Because a total of 15 tests were performed, the resulting *P* values were corrected for multiple comparisons using the Benjamini–Hochberg false discovery rate (FDR) procedure. Follow-up simple effects analyses were performed to clarify the source of the interactions. Paired-samples *t*-tests were used to compare RS vs. MI-BCI within each group; Independent-samples *t*-tests were used to compare UWS vs. MCS within each condition; For each frequency band, the four simple effects were adjusted using the Benjamini–Hochberg FDR correction. All tests were two-tailed, and FDR-corrected *P* < 0.05 was considered statistically significant.

## 3 Results

### 3.1 Results of the mixed-design analysis of variance for each EEG frequency band and DTABR

The main effect of Condition was significant across all frequency bands, displaying big differences between the RS and MI-BCI conditions. The main effect of Group was also significant across all bands, reflecting systematic differences within spectral power between UWS and MCS patients. Significant Group × Condition interactions were identified in the gamma, delta, and beta bands, suggesting that condition-related changes varied between groups within these frequency ranges. For DTABR, the analysis demonstrated a strong main effect of Condition, with DTABR significantly higher in the RS condition than in the MI-BCI condition. The Group × Condition interaction was also significant, indicating that the effect of condition varied between groups. Descriptively, DTABR was higher in MCS than in UWS under RS, while the pattern was reversed under MI-BCI. The main effect of the group was comparatively small. Particular details are provided in Table 2.

**Table 2:** Main and interaction effects of condition and group within each EEG frequency band.

Band	Effect	*F*	*P*	FDR- corrected *P*	$\eta_{\text{p}}^{2}$
Delta	A	95.42	< 0.001	< 0.001	0.841
	B	2,536.00	< 0.001	< 0.001	0.993
	A × B	24.01	< 0.001	< 0.001	0.571
Theta	A	33.43	< 0.001	< 0.001	0.650
	B	44.01	< 0.001	< 0.001	0.710
	A × B	2.91	0.105	0.113	0.139
Alpha	A	16.88	0.001	0.001	0.484
	B	958.30	< 0.001	< 0.001	0.982
	A × B	1.70	0.208	0.208	0.087
Beta	A	8.27	0.010	0.012	0.315
	B	674.40	< 0.001	< 0.001	0.974
	A × B	13.50	0.002	0.002	0.429
Gamma	A	17.41	0.001	0.001	0.492
	B	202.30	< 0.001	< 0.001	0.918
	A × B	16.98	0.001	0.001	0.485
DTABR	A	1.30	0.27	0.27	0.07
	B	562.30	< 0.001	0.003	0.97
	A × B	8.18	0.01	0.015	0.31

(A) Group: UWS vs. MCS; (B) Condition: RS vs. MI-BCI.

### 3.2 Results of a simple effect analysis for the delta, beta, gamma bands and DTABR

**Figure 2:**
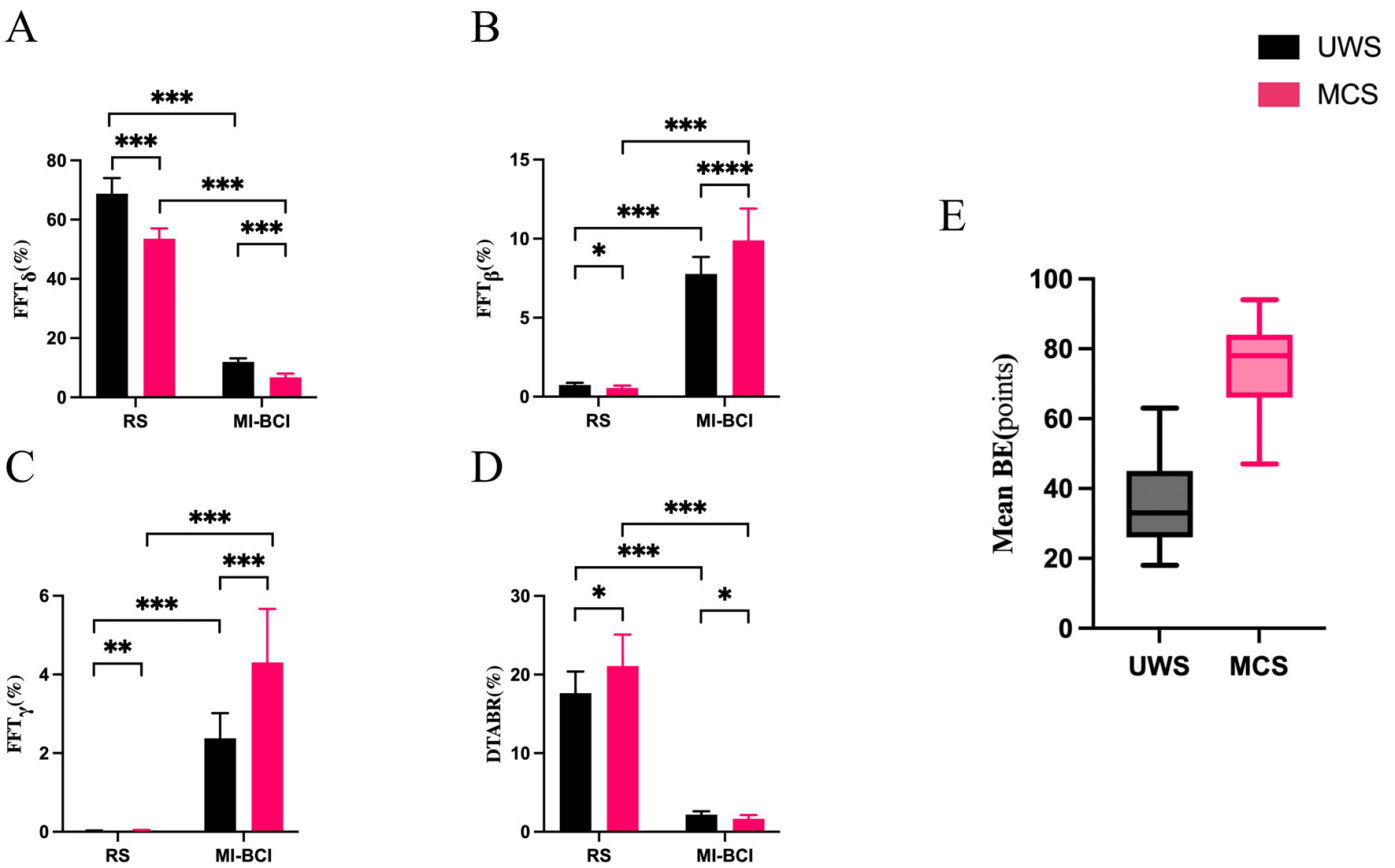
**(A-C)** Relative power in delta, beta, and gamma bands for UWS and MCS patients during resting and MI-BCI task. **(D)** Relative power ratios in UWS and MCS patients during resting and MI-BCI task. **(E)** Average BE during MI-BCI task. Asterisks denote statistical significance: *P < 0.05, **P < 0.01, ***P < 0.001.

Because significant Group × Condition interactions were observed in the gamma, delta, beta bands, and DTABR, simple effects analyses were conducted to probe the source of the interactions. In the delta band, both groups showed significant condition effects, with RS power higher than MI-BCI. Moreover, UWS exhibited significantly higher delta power than MCS under both RS and MI-BCI conditions. In the beta band, significant condition effects were also found in both groups, with MI-BCI power higher than RS. Further group comparisons indicated that UWS was higher than MCS under RS, whereas MCS was higher than UWS under MI-BCI, suggesting a direction-reversing interaction pattern. In the gamma band, significant condition effects were observed within both groups, with MI-BCI showing higher power than RS in both UWS and MCS. In addition, group differences were significant under both RS and MI-BCI conditions, with MCS exhibiting higher gamma power than UWS, and the between-group difference was more pronounced under MI-BCI. In the DTABR, which was significantly higher in RS than in MI-BCI within both UWS and MCS groups. In addition, the group difference was significant under both conditions. MCS showed higher DTABR than UWS under RS, whereas UWS showed higher DTABR than MCS under MI-BCI. Details are shown in Figures 2A–D.

### 3.3 Comparison of average BE between two patient groups during MI-BCI task

During MI-BCI task, patients in the MCS group exhibited higher average BE than those in the UWS group, as shown in Figure 2E.

## 4 Discussion

The results of this study indicate that both the level of consciousness in pDoC patients and whether they performed the MI-BCI task exerted a certain influence on quantitative EEG outcomes, with an interaction effect observed between the two factors. During MI-BCI task performance, both MCS and UWS patients exhibited reduced FFTδ and DTABR compared to the resting state, while FFTα, FFTβ, and FFTγ increased. This indicates that MI-BCI-based virtual reality stimulation modifies EEG response patterns (Al Boustani et al., 2022). Extensive research has long demonstrated that EEG characteristics in patients with impaired consciousness reflect changes in overall brain functional status and consciousness levels. Quantitative EEG in patients with impaired consciousness typically shows significantly increased delta and theta band power, along with reduced alpha and beta band power. This may correlate with diminished brain metabolism or impaired cortical-thalamic network function, suggesting compromised higher cognitive abilities. Furthermore, reduced or absent high-frequency gamma oscillations correlate with impaired integration of conscious content (Beuchat et al., 2022; Zheng et al., 2021). Quantitative EEG studies across different levels of consciousness reveal that patients in the Unconscious State (UWS) exhibit lower alpha and theta band power than those in the MCS, while displaying higher delta band power (Piarulli et al., 2016). This aligns with the RS quantitative EEG characteristics observed in patients with pDoC in the present study.

Concurrently, this study found that RS DTABR was elevated in both groups compared to MI-BCI states, with UWS patients exhibiting higher DTABR than MCS patients during MI-BCI. This reflects the overall trend of reduced slow waves and increased fast waves in the EEG during MI-BCI task, with more pronounced changes in MCS patients. However, previous studies on DTABR in patients with impaired consciousness are limited. Some relevant research indicates that the DTABR values of individuals with impaired consciousness showed a negative correlation with CRS-R scores, suggesting that this indicator can reflect differences in the consciousness state gradient (Wu et al., 2018). Furthermore, elevated DTABR in acute ischemic stroke patients serves as an independent predictor of poor prognosis, while in patients with mild cognitive impairment and Alzheimer's disease, increased DTABR correlates with declining MMSE scores (Niu et al., 2024). This suggests that DTABR changes may represent a potential biomarker for prognosis and cognitive impairment severity in patients with impaired consciousness.

Previous studies have demonstrated that during motor imagery in healthy individuals, the power spectral density of the mu and beta bands in the sensorimotor cortex significantly decreases, resulting in event-related desynchronization. This inhibition reflects neural activation associated with motor preparation and imagery, while alpha-rhythm desynchronization in the primary motor cortex correlates with motor imagery (Chen et al., 2021; Putzolu et al., 2022). However, due to limited cognitive abilities, quantitative EEG characteristics during MI-BCI task developed for healthy individuals remain unclear in pDoC patients. In this study, we observed that most UWS patients struggled to initiate or sustain MI-BCI task, whereas MCS patients showed greater engagement and higher task completion rates. EEG analysis further revealed that MCS patients exhibited higher FFTβ and FFTγ than UWS patients, while FFTδ and DTABR were lower. This indicates distinct EEG response patterns during MI-BCI task across pDoC patients with varying levels of consciousness. Research indicates that BCI can bypass behavioral response limitations by directly detecting brain activity associated with external stimuli, enabling more sensitive identification of residual consciousness in patients (Spataro et al., 2022). In MCS patients, motor imagery tasks can induce event-related desynchronization in characteristic frequency bands similar to those of healthy individuals, suggesting that MCS patients retain some cortical activation capacity (Curley et al., 2018). Although studies have detected alpha rhythm event-related desynchronization in the frontal lobe and ipsilateral motor areas during motor imagery in UWS patients (Formaggio et al., 2020), and brief gamma-band energy enhancement or event-related potential components may appear in EEG during upper limb movement imagery (Kondziella and Stevens, 2022), the low responsiveness of EEG signals during motor imagery tasks in UWS patients is often reflected in low BCI decoding accuracy, approximately 18.5% (Pan et al., 2020). This aligns with the MI-BCI task performance and partial EEG analysis results of UWS patients in this study, though further research is needed.

Our MI-BCI state inherently involves visual stimulation from the virtual reality environment and auditory cues. These exogenous stimuli could elicit arousal or startle responses, particularly in a vulnerable population such as pDoC patients. Various sensory stimuli, including auditory and visual stimulation, have been shown to influence EEG characteristics in patients with disorders of consciousness (Jain and Ramakrishnan, 2020). For instance, compared to the resting state, auditory stimulation significantly increases the absolute power spectral density of delta and theta waves in patients with a MCS (Ling et al., 2023), while decreasing the DTABR value (Wu et al., 2018). Although our findings suggest that differential responses between MCS and UWS patients indicate higher-level cognitive processing in the former, it remains challenging to distinguish genuine autonomous motor imagery from passive sensory stimulus responses solely on the basis of qEEG features. This is particularly difficult in the absence of a control condition exposing patients to the virtual reality environment and background music without administering motor imagery instructions. Furthermore, the observed significant interaction effect between consciousness level and task state on various quantitative EEG features warrants careful interpretation. For patients diagnosed with UWS, the MI-BCI State may, to a significant extent, reflect the EEG response to auditory and visual stimulation rather than active motor imagery and volitional neural control. Consequently, the observed interaction effects may not solely stem from differences in neural processing capabilities, but also from fundamental differences in task compliance between the two groups. This underscores a pivotal challenge in evaluating cognitive function in individuals with severe consciousness impairments, suggesting that future research endeavors should strive to delineate active task engagement from passive sensory processing with greater precision.

The phenomenon of alpha-band phase modulation of beta-band amplitude is considered a hallmark of multiscale neural integration during MI (Santamaria and James, 2018). Complex movement imagery induces greater high-frequency beta activity in the parietal-frontal cortex than simple movement imagery (Putzolu et al., 2022). Therefore, this study employed a relatively complex cycling movement imagery task instead of conventional hand-grasping imagery. BE was defined in this study as the integer value obtained by normalizing the energy ratio of the β band to the α band measured at Fp1 during the MI-BCI task. This index was chosen to reflect the relative increase in beta-band activity over alpha-band activity during task participation, based on the general association of beta rhythms with active cognitive and motor processing and alpha rhythms with more inhibited or non-task-dominant cortical states. Some studies suggest that BE is closely linked to a person's level of attention and cognitive function, and is therefore defined as an attention index (Yuan et al., 2021; Liu et al., 2025). Research on upper-limb MI-BCI has also shown that MCS patients respond better to BCI training, and that BE may be used to assist in distinguishing between UWS and MCS patients (Wan et al., 2025). During the task, the avatar's cycling speed in the virtual reality scene was adjusted based on BE levels, forming a closed-loop feedback mechanism. This enhanced the EEG specificity of the movement imagery response. Results also showed that patients in the MCS group exhibited higher average BE than those in the UWS group. However, in patients with pDoC, alpha activity may be pathologically altered, slowed, or attenuated, which may affect the physiological interpretation of this ratio.

Patients with pDoC frequently experience attention fluctuations, fatigue, and impaired vision or hearing, which present significant challenges for current MI-BCI application research. Observations indicate that pDoC patients have difficulty sustaining prolonged motor imagery tasks. Therefore, the MI-BCI cycling task duration was limited to 5 min to mitigate the effects of attention fluctuations and fatigue. Furthermore, stable EEG signal recording is complicated by patient eye movements and electromyographic interference, necessitating noise suppression through spatial filtering or deep learning algorithms (Lv et al., 2025). Significant individual differences among subjects may also shift effective frequency bands during motor imagery, requiring adaptive band selection to improve classification accuracy (Huang et al., 2024). Decoding EEG information for MI tasks of varying complexity further requires integration of time-frequency and spatial-domain features to enhance specificity (Yang et al., 2024).

In summary, patients with pDoC at different levels of consciousness demonstrate varying capacities for motor imagery, and quantitative EEG features during MI-BCI task also differ accordingly. These findings suggest that MI-BCI holds promise as a diagnostic or evaluative tool for assessing the level of consciousness and residual cognitive capacity in this challenging population. However, this study is limited by the absence of a reverse validation trial to confirm the accuracy of MI-BCI in assessing consciousness levels. The duration of illness among the included pDoC participants varies widely, which may affect their ability to perform motor imagery and their responsiveness to brain-computer interface tasks. Additionally, further investigation of quantitative EEG feature analysis across different brain regions is warranted.

## Data Availability

The original contributions presented in the study are included in the article/supplementary material, further inquiries can be directed to the corresponding author.

## References

[B1] Al Boustani G. Weiß L. J. K. Li H. Meyer S. M. Hiendlmeier L. Rinklin P. . (2022). Influence of auditory cues on the neuronal response to naturalistic visual stimuli in a virtual reality setting. Front. Hum. Neurosci. 16:809293. doi: 10.3389/fnhum.2022.809293 35721351 PMC9201822

[B2] Angerhöfer C. Colucci A. Vermehren M. Hömberg V. Soekadar S. R. (2021). Post-stroke rehabilitation of severe upper limb paresis in Germany - toward long-term treatment with brain-computer interfaces. Front. Neurol. 12:772199. doi: 10.3389/fneur.2021.772199 34867760 PMC8637332

[B3] Beuchat I. Rossetti A. O. Novy J. Schindler K. Rüegg S. Alvarez V. . (2022). Continuous versus routine standardized electroencephalogram for outcome prediction in critically ill adults: analysis from a randomized trial. Crit. Care Med. 50, 329–334. doi: 10.1097/CCM.0000000000005311 34582427 PMC8797015

[B4] Chen Y. Y. Lambert K. J. M. Madan C. R. Singhal A. (2021). Mu oscillations and motor imagery performance: a reflection of intra-individual success, not inter-individual ability. Hum. Mov. Sci. 78:102819. doi: 10.1016/j.humov.2021.102819 34051665

[B5] Curley W. H. Forgacs P. B. Voss H. U. Conte M. M. Schiff N. D. (2018). Characterization of EEG signals revealing covert cognition in the injured brain. Brain 141, 1404–1421. doi: 10.1093/brain/awy070 29562312 PMC5917770

[B6] Formaggio E. Del Felice A. Cavinato M. Storti S. F. Arcaro C. Turco C. . (2020). EEG to identify attempted movement in unresponsive wakefulness syndrome. Clin. EEG Neurosci. 51, 339–347. doi: 10.1177/1550059420911525 32248697

[B7] Giacino J. T. Katz D. I. Schiff N. D. Whyte J. Ashman E. J. Ashwal S. . (2018). Practice guideline update recommendations summary: disorders of consciousness. Neurology 91, 450–460. doi: 10.1212/WNL.0000000000005926 30089618 PMC6139814

[B8] Helbok R. Rass V. Beghi E. Bodien Y. G. Citerio G. Giacino J. T. . (2022). The curing coma campaign international survey on coma epidemiology, evaluation, and therapy (COME TOGETHER). Neurocrit. Care 37, 47–59. doi: 10.1007/s12028-021-01425-8 35141860 PMC9283177

[B9] Huang W. Liu X. Yang W. Li Y. Sun Q. Kong X. . (2024). Motor imagery EEG signal classification using distinctive feature fusion with adaptive structural LASSO. Sens 24:3755. doi: 10.3390/s24123755 38931540 PMC11207242

[B10] Jain R. Ramakrishnan A. G. (2020). Electrophysiological and neuroimaging studies - during resting state and sensory stimulation in disorders of consciousness: a review. Front. Neurosci. 14:555093. doi: 10.3389/fnins.2020.555093 33041757 PMC7522478

[B11] Kondziella D. Stevens R. D. (2022). Classifying disorders of consciousness: past, present, and future. Semin. Neurol. 42, 239–248. doi: 10.1055/a-1883-1021 35738291

[B12] Ling Y. Xu C. Wen X. Li J. Gao J. Luo B. . (2023). Cortical responses to auditory stimulation predict the prognosis of patients with disorders of consciousness. Clin. Neurophysiol. 153, 11–20. doi: 10.1016/j.clinph.2023.06.002 37385110

[B13] Liu P. Ge Q. Dong L. Jiao L. Han S. Kang X. . (2025). Motor imagery-based brain-computer interface for differential diagnosis in prolonged disorders of consciousness. Front. Hum. Neurosci. 19:1695730. doi: 10.3389/fnhum.2025.1695730 41394940 PMC12696706

[B14] Lv R. Chang W. Yan G. Nie W. Zheng L. Guo B. . (2025). A novel recognition and classification approach for motor imagery based on spatio-temporal features. IEEE J. Biomed. Health. Inform. 29, 210–223. doi: 10.1109/JBHI.2024.3464550 39374272

[B15] Niu X. Wang Y. Zhang X. Wang Y. Shao W. Chen L. . (2024). Quantitative electroencephalography (qEEG), apolipoprotein a-I (APOA-I), and apolipoprotein epsilon 4 (APOE ε4) alleles for the diagnosis of mild cognitive impairment and alzheimer's disease. Neurol. Sci. 45, 547–556. doi: 10.1007/s10072-023-07028-9 37673807

[B16] Olivia V. R. Chira D. Chelaru V-. F. Diana C. D. Livia L. P. Buruiană A-. M. . (2024). QEEG indices in traumatic brain injury - insights from the CAPTAIN RTMS trial. J. Med. Life 17, 318–325. doi: 10.25122/jml-2024-0187 39044922 PMC11262599

[B17] Pan J. Xie Q. Qin P. Chen Y. He Y. Huang H. . (2020). Prognosis for patients with cognitive motor dissociation identified by brain-computer interface. Brain: J. Neurol. 143, 1177–1189. doi: 10.1093/brain/awaa026 PMC7174053 32101603

[B18] Piarulli A. Bergamasco M. Thibaut A. Cologan V. Gosseries O. Laureys S. . (2016). EEG ultradian rhythmicity differences in disorders of consciousness during wakefulness. J. Neurol. 263, 1746–1760. doi: 10.1007/s00415-016-8196-y 27294259

[B19] Putzolu M. Samogin J. Cosentino C. Mezzarobba S. Bonassi G. Lagravinese G. . (2022). Neural oscillations during motor imagery of complex gait: an HdEEG study. Sci. Rep. 12:4314. doi: 10.1038/s41598-022-07511-x 35279682 PMC8918338

[B20] Rodríguez-García M. E. Carino-Escobar R. I. Carrillo-Mora P. Hernandez-Arenas C. Ramirez-Nava A. G. Pacheco-Gallegos M. D. R. . (2025). Neuroplasticity changes in cortical activity, grey matter, and white matter of stroke patients after upper extremity motor rehabilitation via a brain-computer interface therapy program. J. Neural Eng. 22:20. doi: 10.1088/1741-2552/adbebf 40064104

[B21] Santamaria L. James C. (2018). Using brain connectivity metrics from synchrostates to perform motor imagery classification in EEG-based BCI systems. Healthc. Technol. Lett. 5, 88–93. doi: 10.1049/htl.2017.0049 29922477 PMC5998754

[B22] Shao H. Deng W. Du R. Zhao Y. Jin D. Wei Y. . (2025). Mismatch negativity and P300 in the diagnosis and prognostic assessment of coma and other disorders of consciousness. Neurocrit. Care 42, 185–195. doi: 10.1007/s12028-024-02058-3 39043983

[B23] Spataro R. Xu Y. Xu R. Mandalà G. Allison B. Z. Ortner R. . (2022). How brain-computer interface technology may improve the diagnosis of the disorders of consciousness: a comparative study. Front. Neurosci. 16:959339. doi: 10.3389/fnins.2022.959339 36033632 PMC9404379

[B24] Wan C. Zhang Q. Qiu Y. Zhang W. Nie Y. Zeng S. . (2025). Effects of dual-task mode brain-computer interface based on motor imagery and virtual reality on balance and attention in patients with stroke: a randomized controlled pilot trial. J. Neuroeng. Rehabil. 22:187. doi: 10.1186/s12984-025-01730-9 40883792 PMC12395916

[B25] Wang J. Hu X. Hu Z. Sun Z. Laureys S. Di H. . (2020). The misdiagnosis of prolonged disorders of consciousness by a clinical consensus compared with repeated coma-recovery scale-revised assessment. BMC Neurol. 20:343. doi: 10.1186/s12883-020-01924-9 32919461 PMC7488705

[B26] Wolpaw J. R. Millán J. D. R. Ramsey N. F. (2020). Brain-computer interfaces: definitions and principles. Handb. Clin. Neurol. 168, 15–23. doi: 10.1016/B978-0-444-63934-9.00002-0 32164849

[B27] Wu M. Bao W.-X. Zhang J. Hu Y.-F. Gao J. Luo B.-Y. . (2018). Effect of acoustic stimuli in patients with disorders of consciousness: a quantitative electroencephalography study. Neural Regener. Res. 13, 1900–1906. doi: 10.4103/1673-5374.238622 30233062 PMC6183039

[B28] Yang K. Li R. Xu J. Zhu L. Kong W. Zhang J. . (2024). DSFE: Decoding EEG-based finger motor imagery using feature-dependent frequency, feature fusion and ensemble learning. IEEE J. Biomed. Health Inform. 28, 4625–4635. doi: 10.1109/JBHI.2024.3395910 38709613

[B29] Yuan Z. Peng Y. Wang L. Song S. Chen S. Yang L. . (2021). Effect of BCI-controlled pedaling training system with multiple modalities of feedback on motor and cognitive function rehabilitation of early subacute stroke patients. IEEE Trans. Neural Syst. Rehabil. Eng. 29, 2569–2577. doi: 10.1109/TNSRE.2021.3132944 34871175

[B30] Zheng Y. Wu S. Yang Q. Xu Z. Zhang S. Fan S. . (2021). Trigeminal nerve electrical stimulation: an effective arousal treatment for loss of consciousness. Brain Res. Bull. 169, 81–93. doi: 10.1016/j.brainresbull.2021.01.008 33453332

